# Supplementary material for: Liquid sculpture and curing of bio-inspired polyelectrolyte aqueous two-phase systems
Source: Nat Commun. 2023 Apr 28;14:2456. doi: 10.1038/s41467-023-38236-8 (PMC10147642; doi:10.1038/s41467-023-38236-8)
Supplement: Supplementary file 1 — Supplementary Information [file 41467_2023_38236_MOESM1_ESM.pdf]

Supplementary Information

Liquid Sculpture and Curing of Bio-inspired Polyelectrolyte Aqueous Two-Phase Systems

Chongrui Zhang, Xufei Liu, Jiang Gong, Qiang Zhao[*]

Key Laboratory of Material Chemistry for Energy Conversion and Storage, (Ministry of Education), School of Chemistry and Chemical Engineering, Huazhong University of Science and Technology, Wuhan 430074, P.R. China. E-mail: zhaoq@hust.edu.cn (Q.Z.)

1.. Supplementary methods

1.1. *Materials:* 1-Vinyl imidazole (99%), 2-(dimethylamino)ethyl methacrylate (98%), bromoacetonitrile ($BrCH_2CN$) (99%), 4-bromobutanenitrile ($Br(CH_2)_3CN$) (98%), 5-bromovaleronitrile ($Br(CH_2)_4CN$) (98%) and bromoethane (98%) were purchased from Aladdin Industrial Co., Ltd., Shanghai, China. Methacrylatoethyl trimethyl ammonium chloride (TMAC) (98%) was obtained from Shanghai Titan Scientific Co., Ltd, Shanghai, China. 2'-Azobis(isobutyronitrile) (AIBN) (99%) was purchased from Fuchen Chemical Reagents Co., Ltd., Tianjin, China. Dimethyl sulfoxide (DMSO), tetrahydrofuran (THF) and sodium hydroxide (NaOH) were analytical reagents. All raw materials were used as purchased.

1.2. *Synthesis of IL monomers:* Synthesis of ILCN1: 1-Vinyl imidazole (10 g, 0.11 mol) and $BrCH_2CN$ (16.5g, 0.14 mol) were dissolved in 150 mL THF and added into a round bottom flask. The mixture was heated in oil bath at 60 °C for 12 h. The white precipitates, 1-cyanomethyl-3-vinylimidazolium bromide (ILCN1) were filtered and washed with THF for 3 times and vacuum dried at 50 °C for 12 h. 1-cyanopropyl-3-vinylimidazolium bromide (ILCN2) was synthesized through the reaction of 1-vinyl imidazole with $Br(CH_2)_3CN$ at the same condition of ILCN1. 1-cyanobutyl-3-vinylimidazolium bromide (ILCN3) was synthesized through the reaction of 1-vinyl imidazole with $Br(CH_2)_4CN$ at the same condition of ILCN1. N-cyanomethyl-N-methacryloxyly-N, N-dimethyl ammonium bromide (ILCN4) was synthesized through the reaction of 2-(dimethylamino)ethyl methacrylate with $BrCH_2CN$ under the same condition of ILCN1. 1-ethyl-3-vinylimidazolium bromide (ILeth) was synthesized

through the reaction of 1-vinyl imidazole with bromoethane at the same condition of ILCN1. Chemical structures of all monomers were characterized by ^1H NMR (Supplementary Fig. 1), in which all characteristic peaks were well assigned.

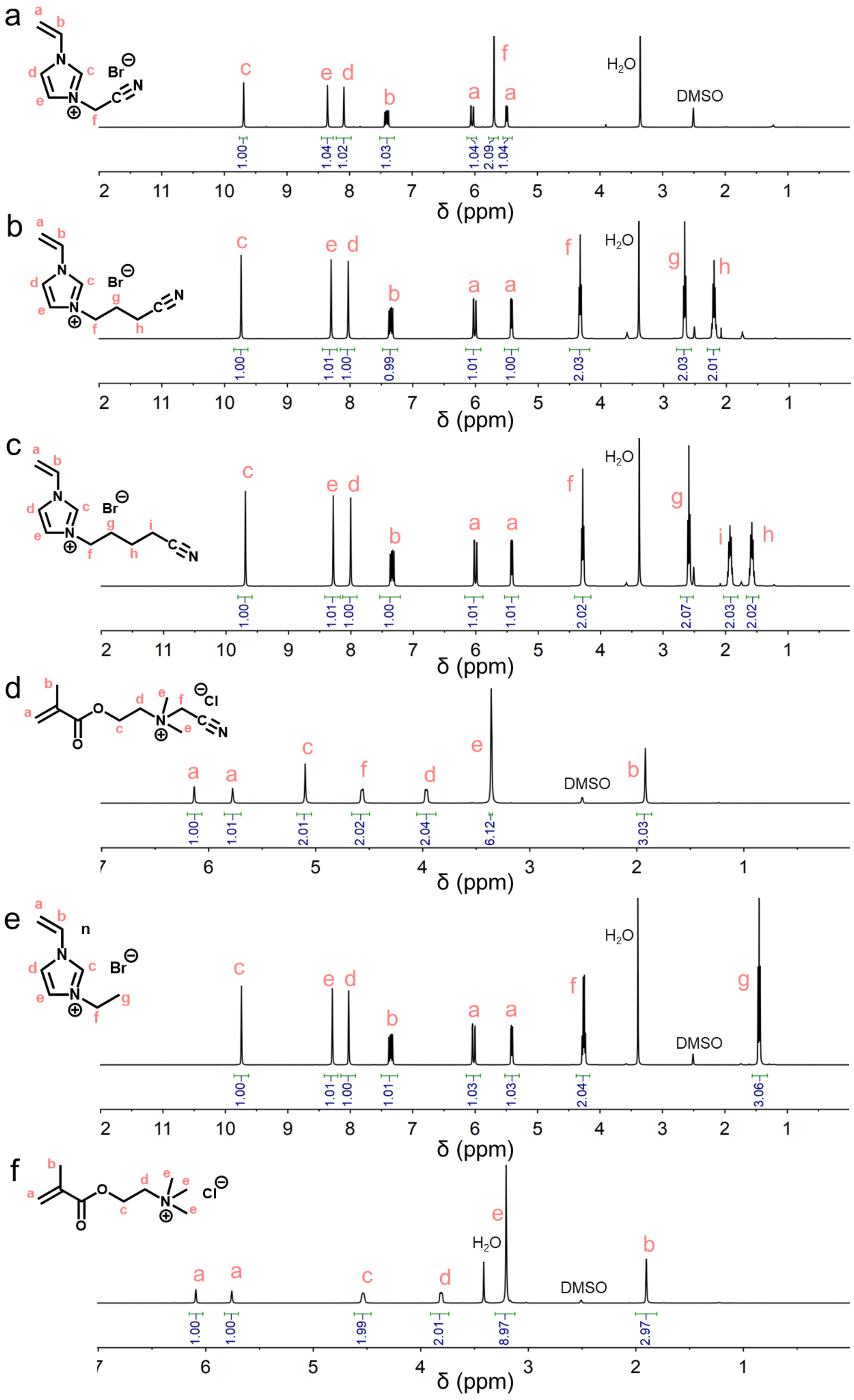

Supplementary Fig. 1. ^{1}H-NMR spectrum of monomers: (a) ILCN1, (b) ILCN2, (c) ILCN3, (d) ILCN4, (e) ILeth and (f) TMAC in DMSO-d6.

1.3. *Synthesis of PILs:* IL monomers (ILCN1, ILCN2, ILCN3, ILCN4, ILeth and TMAC) (10 g), AIBN (1 mol% of monomer) and DMSO (100 mL) were added into a flask equipped with a magnetic stirrer. The mixture was deoxygenated three times by a freeze-pumpthaw procedure, refilled with nitrogen. Afterwards the solution was heated at 80 ℃ for 12 h, cooled to room temperature. And the solution was dropped into excessive THF (1 L), and the precipitates PILs (PILCN1, PILCN2, PILCN3, PILCN4, PILeth and PTMAC) were collected, washed with THF three times and vacuum dried at 50 ℃ for 12 h.

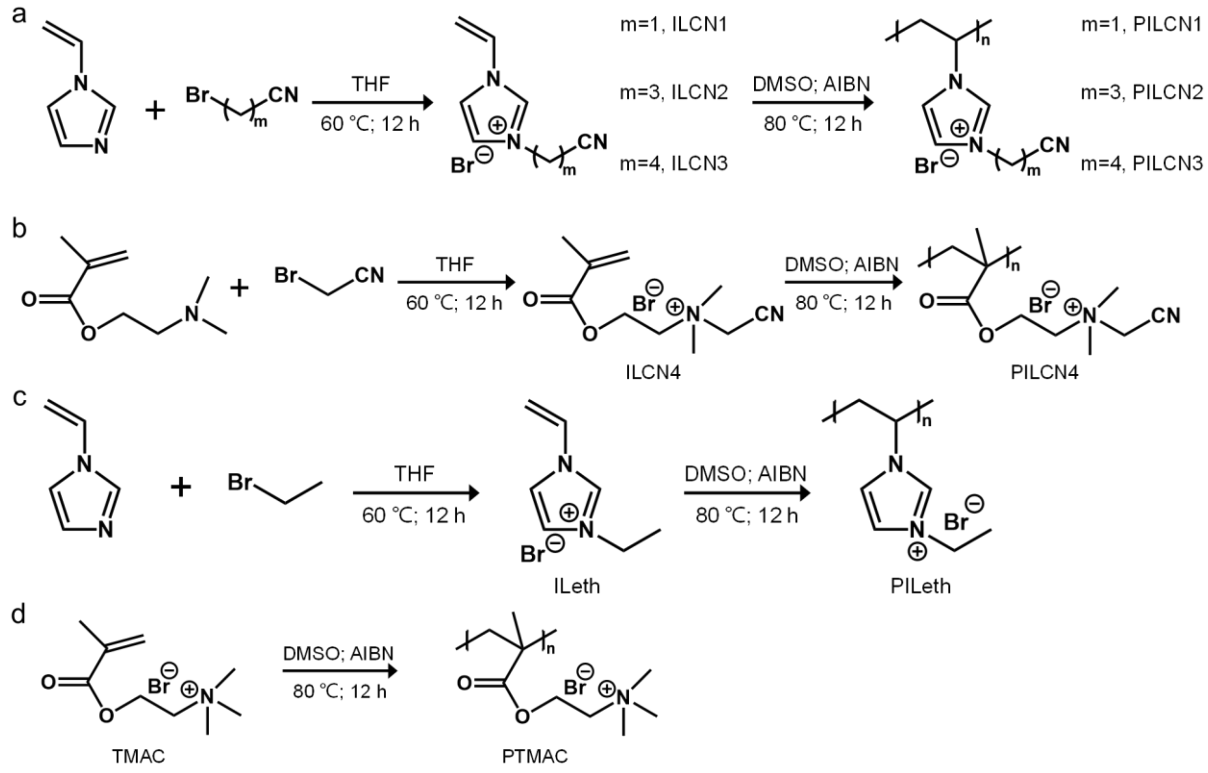

Supplementary Fig. 2. Synthesis of (a) PILCNx (x=1-3), (b) PILCN4, (c) PILeth and (d) PTMAC.

1.4. *Preparation of PILCN1-PDDA-CNT particles (Fig. 6j, 6k)*: A designed amount of carbon nanotubes (CNTs) were dispersed in both PILCN1 and PDDA solution, and mixed to form ATPS at 20 ^{o}C. The pH value of this ATPS was tuned to 11 without stirring, and the ATPS was kept at pH 11 for 1 h at 20 ^{o}C. Then the ATPS was ultra-sonicated (650 W) for 5 mins, purified by centrifugation (6800 g) and water washing cycle (3 times), freezing dried, and examined by SEM. Please note: a part of microbeads in the ATPS were partially broken during the ultra-sonication, so that the interior part of these particles could be examined by SEM.

1.5. ***Characterizations:*** ^{1}H NMR (Nuclear Magnetic Resonance) spectra were conducted in Bruker Avance III 400MHz. Gel Permeation Chromatography (GPC) analysis was carried out on a PL GPC 50 instrument equipped with 8 μm PL MIXED chromatography columns with water as solvent at 30 °C, and the values were calibrated versus polyethylene glycol standard. Field Emission Scanning Electron Microscopy (FESEM) was done with a SU-8010 equipment. Up-conversion spectral scanning confocal microscopy (Olympus FV1200) was done with 405 nm light excitation. Attenuated total reflectance Fourier transform infrared spectroscopy was obtained by a Bruker Vertex 80. All samples were scanned for three times. The compression measurements were performed with an electronic universal testing machine (UTM2103, China), whereby the CNT-hydrogel was compressed (10 mm min^{-1}) at room temperature. The transmittance, reflection and absorption of the CNT-hydrogel were obtained by a Lambda 750s Vis-NIR Spectrophotometer (PerkinElmer) attached with an integrating sphere. pH values were measured by a digital pH meter (SARTORIUS, pB-10). Organic elemental analysis (C and N) was performed on a Vario EL from Elementar. The compositions of bottom liquid and top liquid (Table 1 and 2, Fig. S6) were calculated by the following equation:

$$C_{PILCN1} = C_{pol}(20544-2996M_{C/N})/(3787M_{C/N}+6978)$$

Above, C_{PILCN1} is the concentration of PILCN1 in bottom or top liquid, C_{pol} is the total polymer concentration of bottom or top liquid, $M_{C/N}$ is the mass ratio of C to N in dried two phases.

1.6. Solar thermal steaming measurements: The solar thermal steaming measurement was conducted by a solar light simulator (CEL-S500L) at 30 °C and 50% relative humidity. Typically, the CNT-hydrogel were put on the surface of a 0.5 cm thick polystyrene foam, which was employed as the thermal insulating layer and wrapped by hydrophilic cotton to facilitate water transport. The water mass change was measured by an electronic balance (JA2003, Soptop). The surface temperature of the CNT-hydrogel was monitored by the infrared camera. The evaporation rate (kg m^{-2} h^{-2}) and solar-to-vapor conversion efficiency were calculated by the following equations:

$$\text{Evaporation rate} = \Delta m/(S * t)$$

Above, Δm is the mass change of water during 1 h evaporation under 1-sun irradiation, S is the

area (m^2) of the material for evaporation, and t represents the time of solar irradiation.

The evaporation enthalpy was calculated by the following two equations:

$E_1 = E_0 * m_0/m_1$

Solar-to-vapor conversion efficiency = $(m_2 - m_1) * E_1/(3600 * P)$

Above, E_1 is the evaporation enthalpy of water on CNT-hydrogel, E_0 is the evaporation enthalpy of pure water at 30 ºC, which is 2429.8 kJ kg^{-1}, m_0 is the mass of water evaporated in the dark (1 h, 30 ºC), m_1 is the mass of water evaporated *via* the CNT-hydrogel in the dark (1 h), m_2 is the mass of water evaporated *via* 1-sun irradiation on CNT-hydrogel (1 h), and P is the irradiation intensity (1 kW m^{-2}).

2. Supplementary Discussions

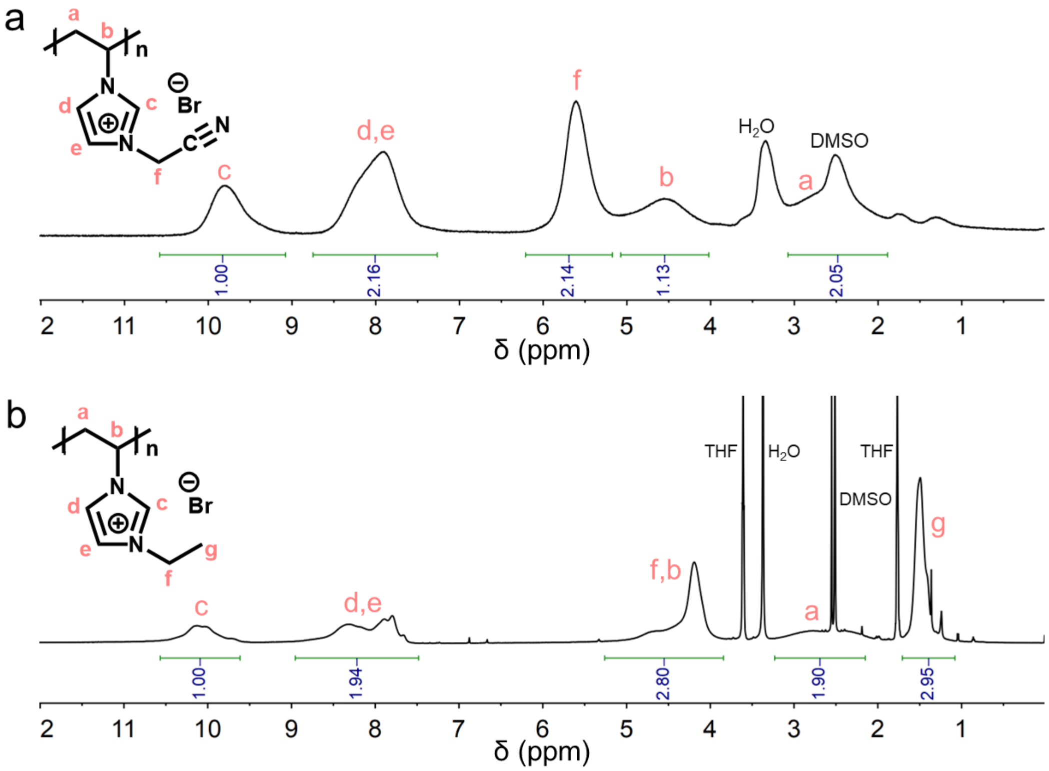

Supplementary Fig. 3. ^{1}H-NMR spectrum of (a) PILCN1 and (b) PILeth in DMSO-d6.

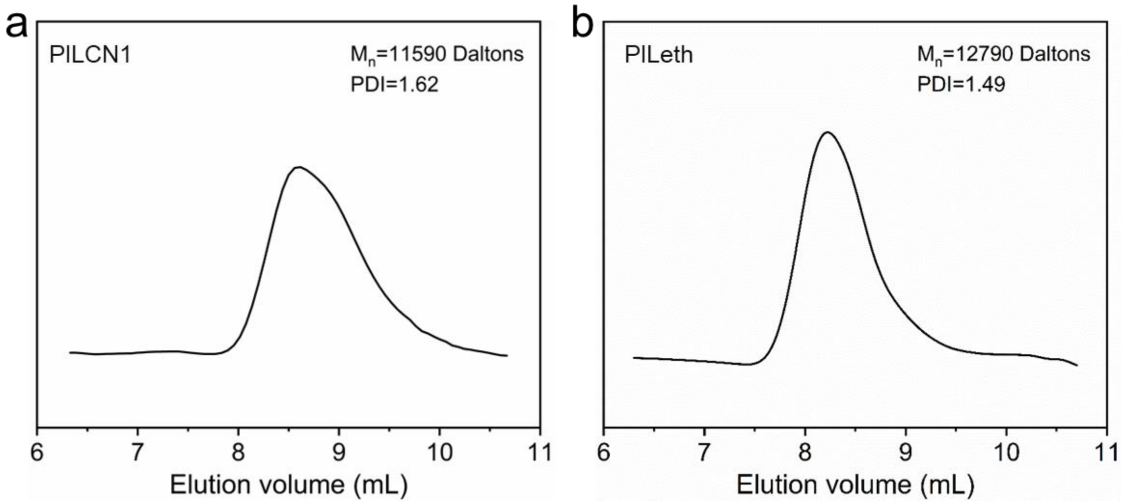

Supplementary Fig. 4. GPC traces of (a) PILCN1 and (b) PILeth (eluent: water). Their apparent number-average molecular weights and polydispersity index (PDI) values were indicated.

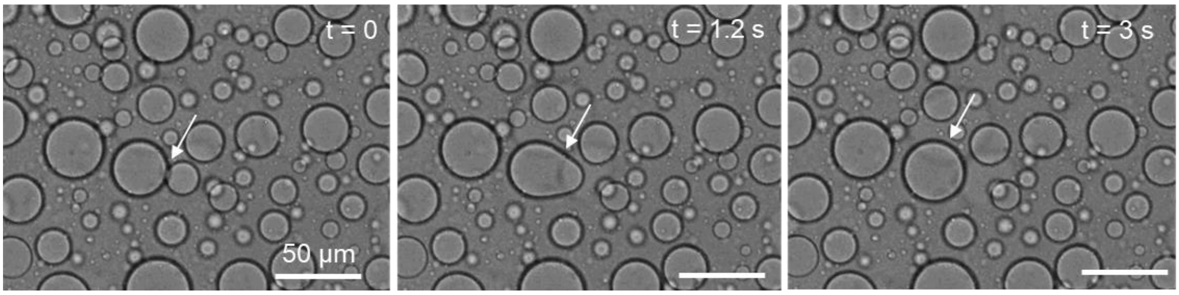

Supplementary Fig. 5. Structure evolution of PILCN1-PDDA mixture observed by optical microscopy. Scale bars are all 50 μm.

Discussion: As noted by arrows, droplets are merging with increasing time. This results indicates the liquid nature of these droplets.

Supplementary Table 1 Polymer concentration and volume fraction of bottom and top phases of PILCN1 (1 mL; 100 mg/mL)-PDDA (1 mL; 50, 60, 80, 100 mg/mL) ATPSs.

PILCN1 /PDDA	Bottom		Top	
	C_{pol} /mg/mL	V/%	C_{pol} /mg/mL	V/%
10/5	274	13.1	45	86.9
10/6	282	13.7	48	86.3
10/8	289	15.7	53	84.3
10/10	293	17.5	59	82.5

Supplementary Table 2 Organic elemental analysis of lyophilized bottom and top phases of PILCN1 (1 mL; 100 mg/mL)-PDDA (1 mL; 50, 60, 80, 100 mg/mL) ATPS.

PILCN1 /PDDA	Bottom			Top		
	C/wt%	N/wt%	$M_{C/N}$	C/wt%	N/wt%	$M_{C/N}$
10/5	39.29	16.09	2.44	40.61	12.08	3.36
10/6	39.33	17.31	2.27	41.45	10.02	4.14
10/8	39.34	18.24	2.16	43.26	8.74	4.95
10/10	40.92	19.50	2.10	45.08	8.33	5.41

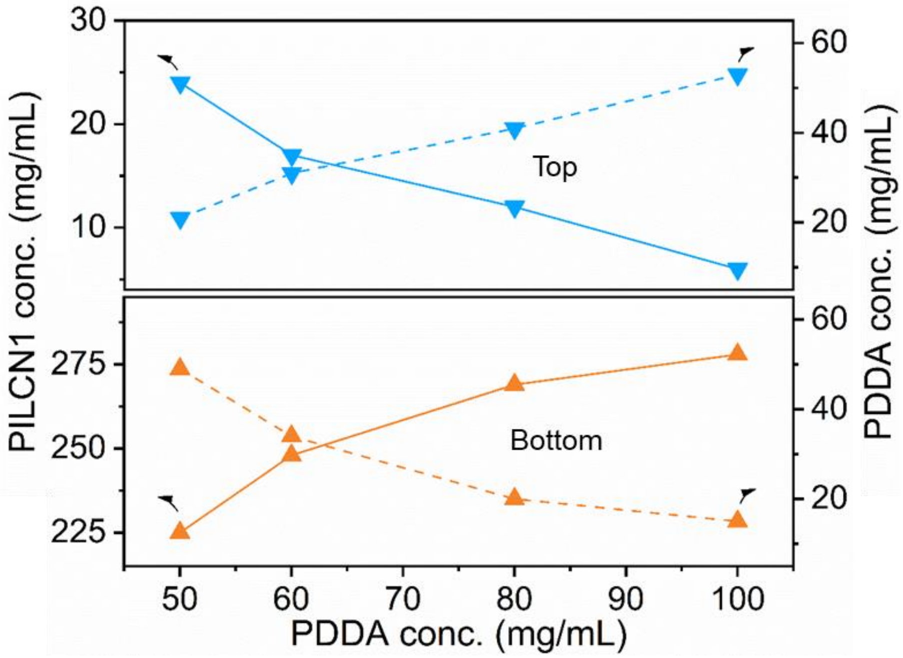

Supplementary Fig. 6. Compositions of bottom and top phases of ATPS. Please note: these ATPS were prepared by mixing PILCN1 (1 mL, 100 mg/mL) solution and PDDA (1 mL, 50 mg/mL-100 mg/mL) solution at 20 ºC, standing for 5-h. Then samples from bottom and top phases were freezing dried, characterized by organic elemental analysis.

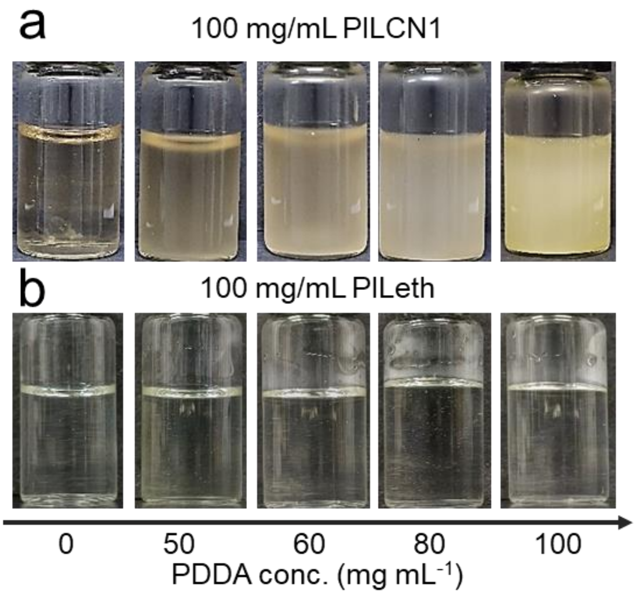

Supplementary Fig. 7. (a) Optical photographs of mixtures of PILCN1 (1 mL; 100 mg/mL) and PDDA (1 mL; 0, 50, 60, 80, 100 mg/mL). (b) Optical photographs of mixtures of PILeth (1 mL, 100 mg/mL) and PDDA (1 mL; 50, 60, 80, 100 mg/mL).

Discussion: (a): turbid were seen when PILCN1 was mixed with PDDA. (b): the PILeth-PDDA mixture remains a homogeneous, transparent solution, indicate that NO ATPS form by mixing PILeth and PDDA.

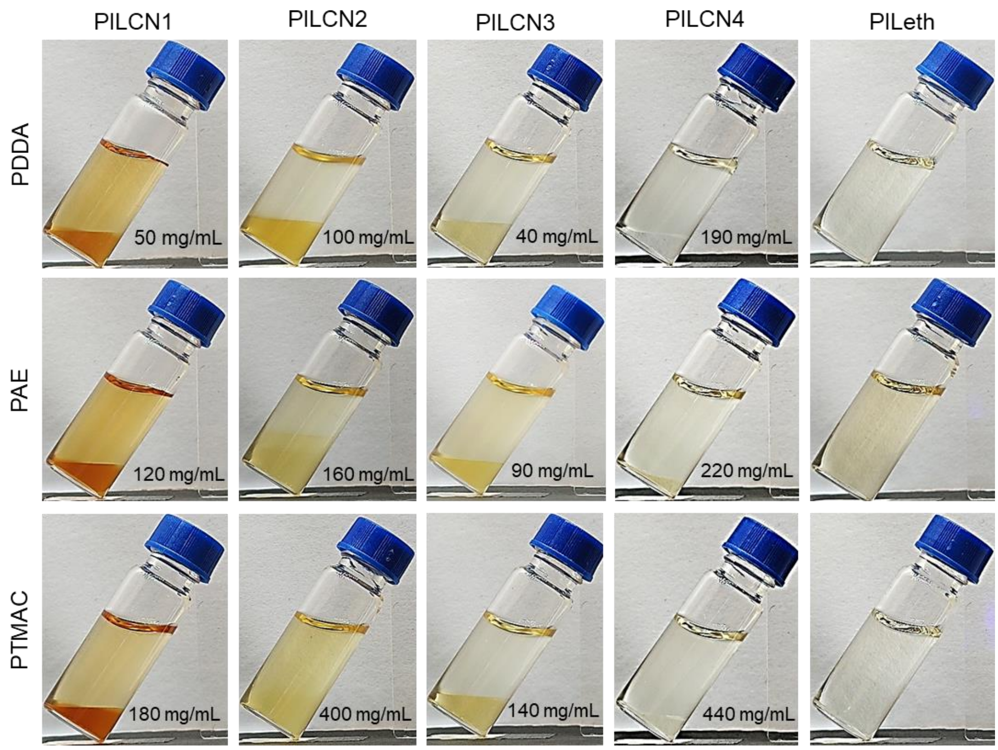

Supplementary Fig. 8. Optical photographs of the PIL (PILCN1-4 or PILeth)-polycations (PDDA, PAE and PTMAC) mixtures after 5-h standing at 20 ºC.

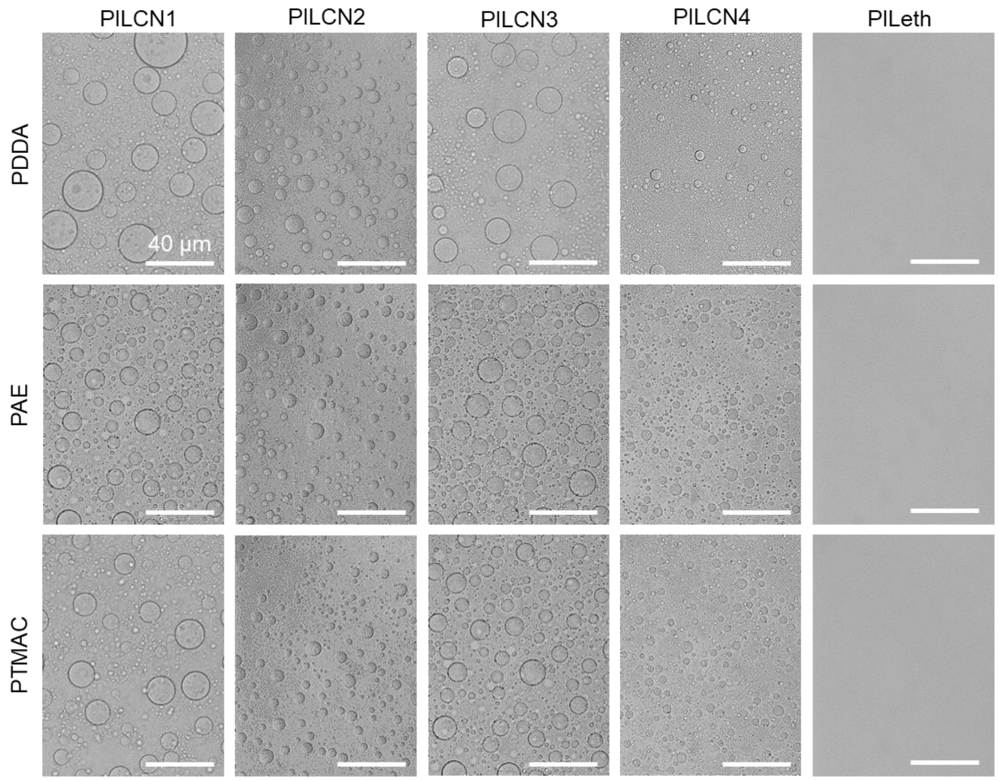

Supplementary Fig. 9. Optical microscopy images of the PIL (PILCN1-4 or PILeth)-polycations (PDDA, PAE and PTMAC) mixtures. Scale bars are all 40 μm.

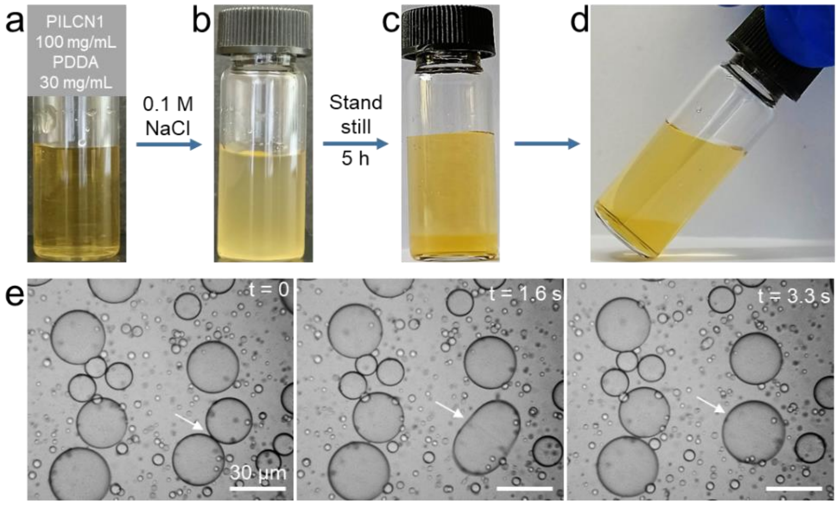

Supplementary Fig. 10. (a) Aqueous solution mixture of PDDA (1 mL, 30 mg/mL) and PILCN1 (1 mL,100 mg/mL); (b) Formation of PILCN1-PDDA ATPS after adding 0.1 M NaCl to (a) at 20 °C; (c,d) Optical photographs of (b) after 5-h standing (c) and being tilted (d); (e) Structure evolution of droplets in (b) with increasing time. Scale bars are all 30 μm.

Discussion: Supplementary Fig. 8a shows that a PILCN1-PDDA homogeneous solution was turned into a dispersion (Supplementary Fig. 8b) by adding NaCl. After 5-h standing, ATPS was observed (Supplementary Fig. 8c, 8d).

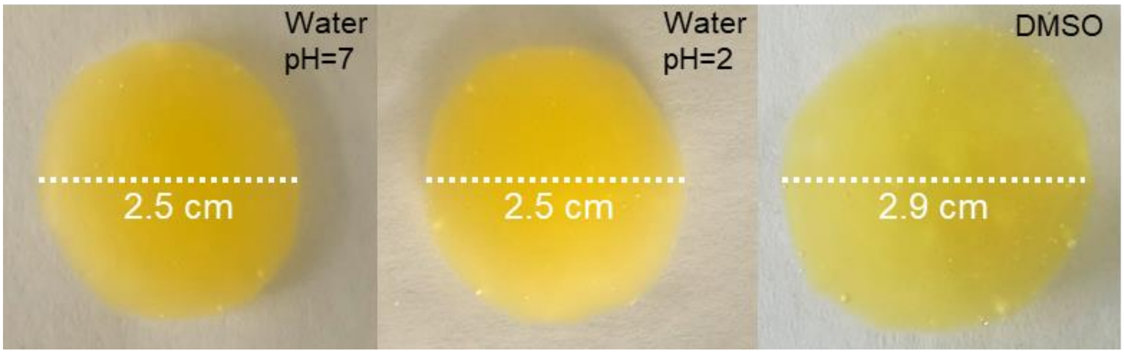

Supplementary Fig. 11. Optical photographs of the PILCN1-PDDA hydrogel soaked in water (pH 2, 7) and DMSO, respectively.

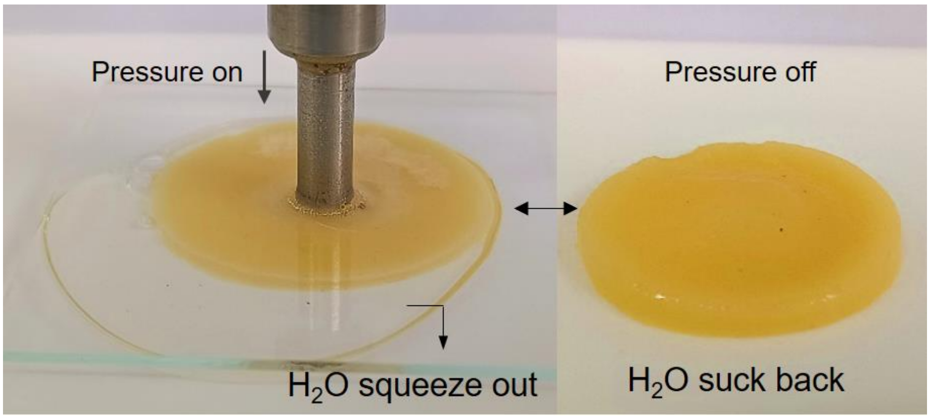

Supplementary Fig. 12. Optical photographs of the hydrogel being reversibly pressed.

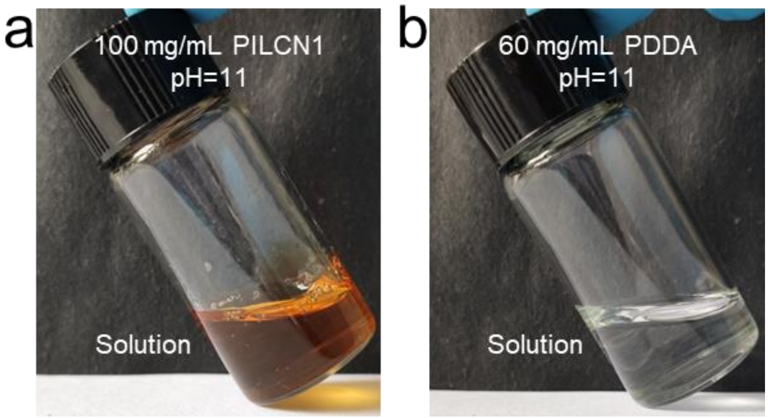

Supplementary Fig. 13. Optical photographs of (a) PILCN1 (100 mg/mL, pH=11) and (b) PDDA (60 mg/mL, pH=11) aqueous solution, respectively.

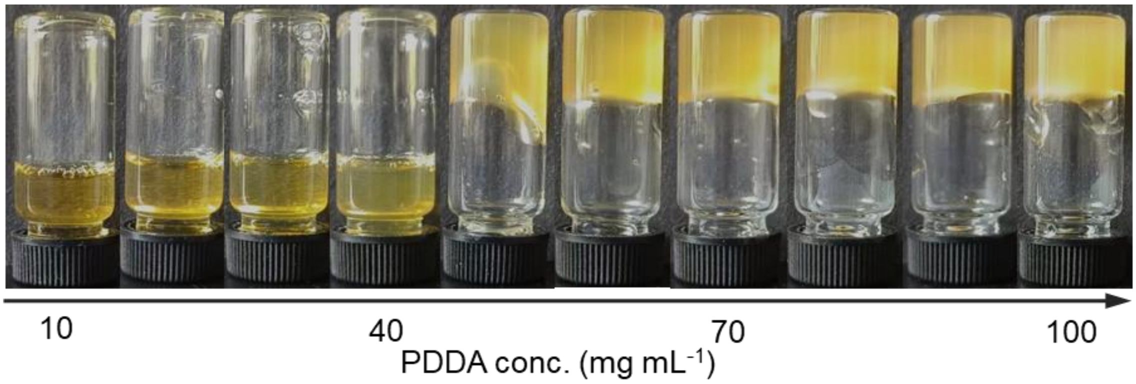

Supplementary Fig. 14. Optical photographs of the mixtures of PILCN1 (1 mL, 100 mg/mL, pH 11) and PDDA (1 mL) with different concentrations (from left to right: 10 ~ 100 mg/mL, pH 11).

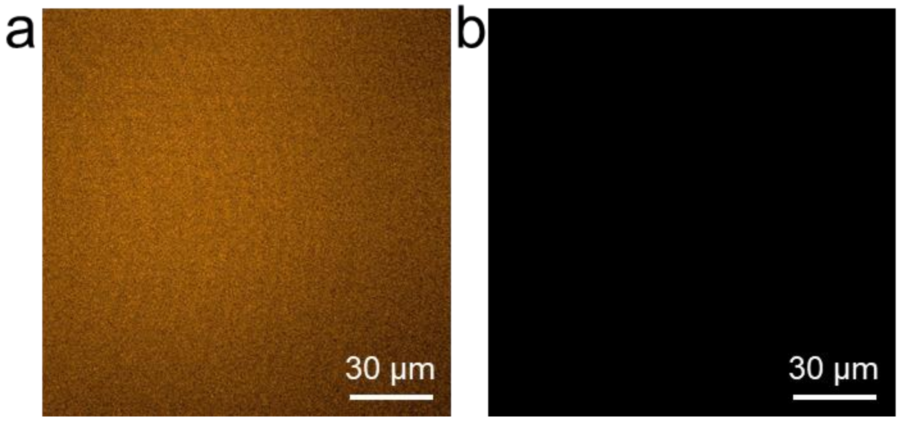

Supplementary Fig. 15. Laser confocal fluorescent microscopy images of (a) PILCN1 (100 mg/mL) and (b) PDDA (60 mg/mL) solution. Please note: 405 nm light excitation.

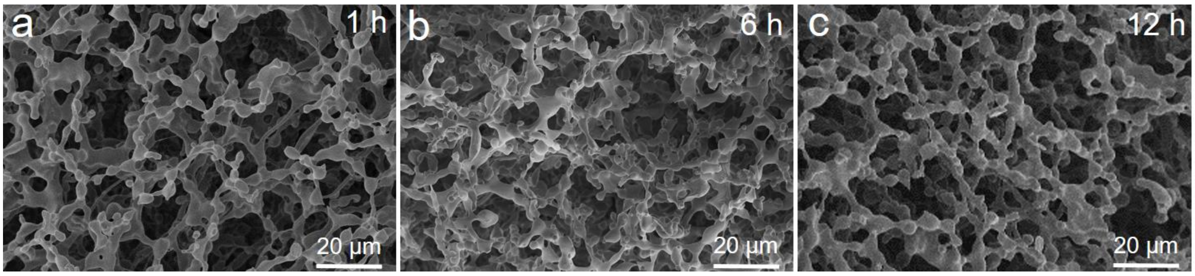

Supplementary Fig. 16. SEM morphologies of hydrogel soaked in water (pH=11) for (a) 1 h, (b) 6 h and (c) 12 h, respectively.

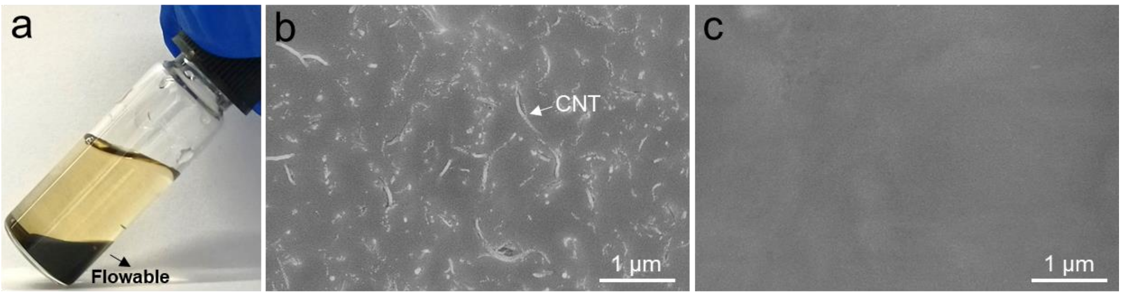

Supplementary Fig. 17. (a) Optical photograph of the layered ATPS containing CNT tilted; SEM images of (b) bottom and (c) top dispersions dried on wafer in (a), respectively.

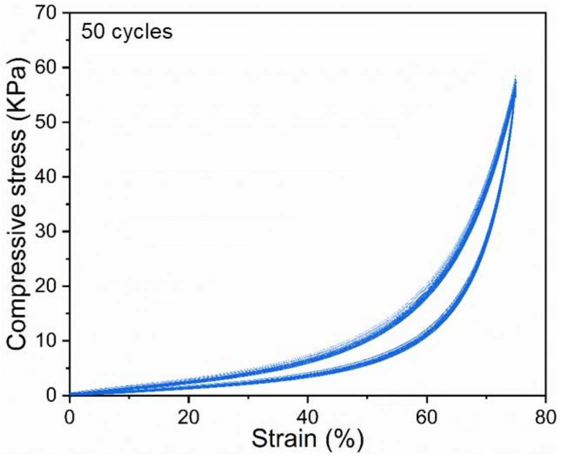

Supplementary Fig. 18. Compressive stress-strain curves of the CNT hydrogel. Please note: CNT content in the hydrogel is 0.65wt%.

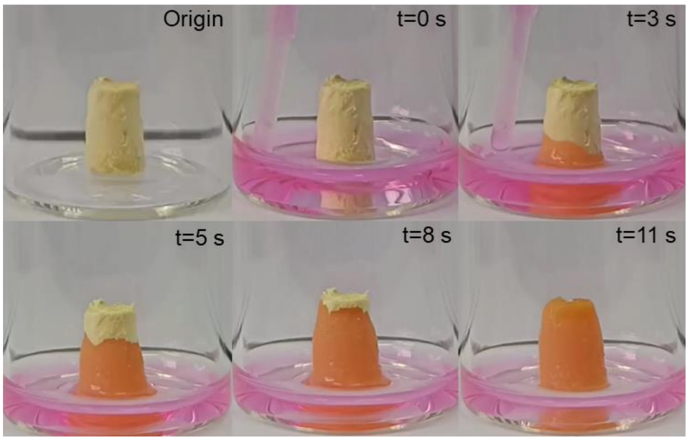

Supplementary Fig. 19. Optical photographs of immersing a freezing-dried PILCN1-PDDA hydrogel (1 cm height) in water containing a trace amount of Rhodamine B dye (for visibility purpose). Please note: In order to improve the visibility of water absorption, CNTs (black color) were not incorporated in the hydrogel.

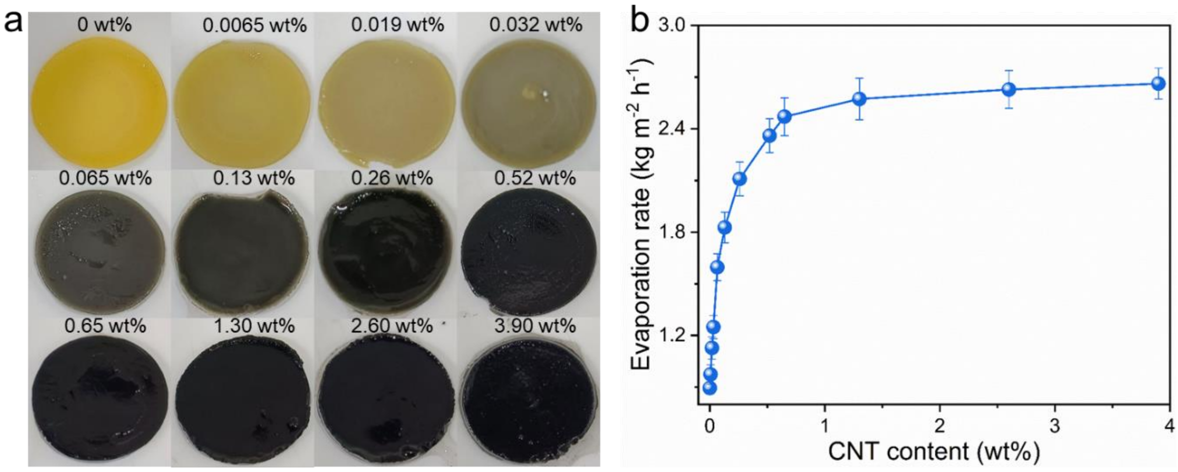

Supplementary Fig. 20. (a) Optical photographs of CNT-hydrogels added with different amount CNTs. (b) Effect of CNT content on the solar thermal evaporation of the CNT-hydrogel under 1-sun irradiation. Note: error bars in (b) are standard deviations of evaporation rate of CNT-hydrogels.

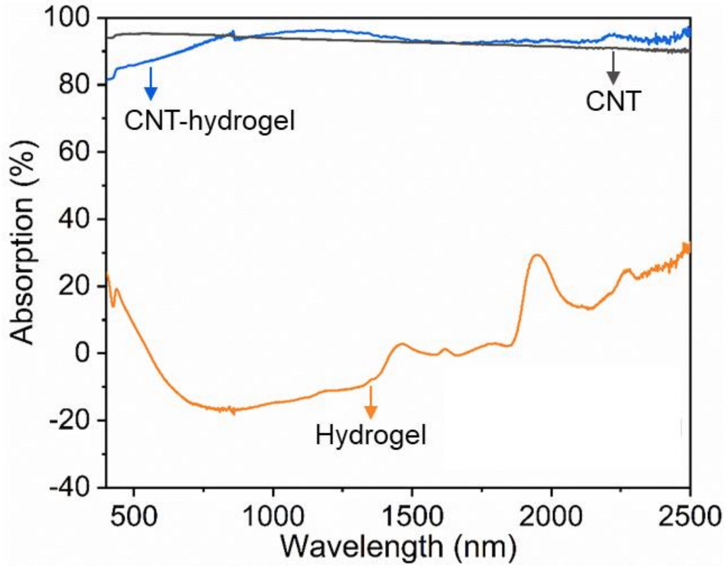

Supplementary Fig. 21. Absorption of different materials in the UV-Vis-NIR regions.

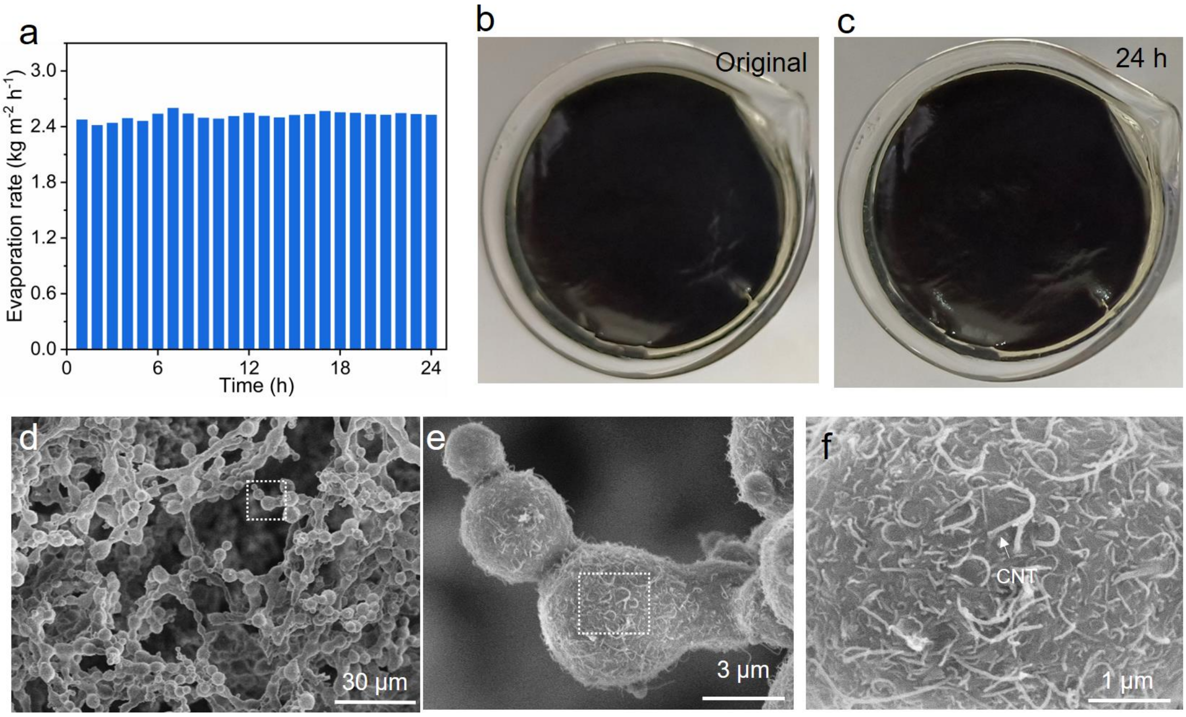

Supplementary Fig. 22. (a) Effect of running time in water evaporation rate of CNT-hydrogel with 0.65 wt% CNT under 1-sun irradiation, (b,c) optical photographs of the CNT-hydrogel evaporator before and after 24 h solar thermal operation in (a), (d-f) SEM micrographs of the CNT-hydrogel evaporator after 24-h solar thermal operation.

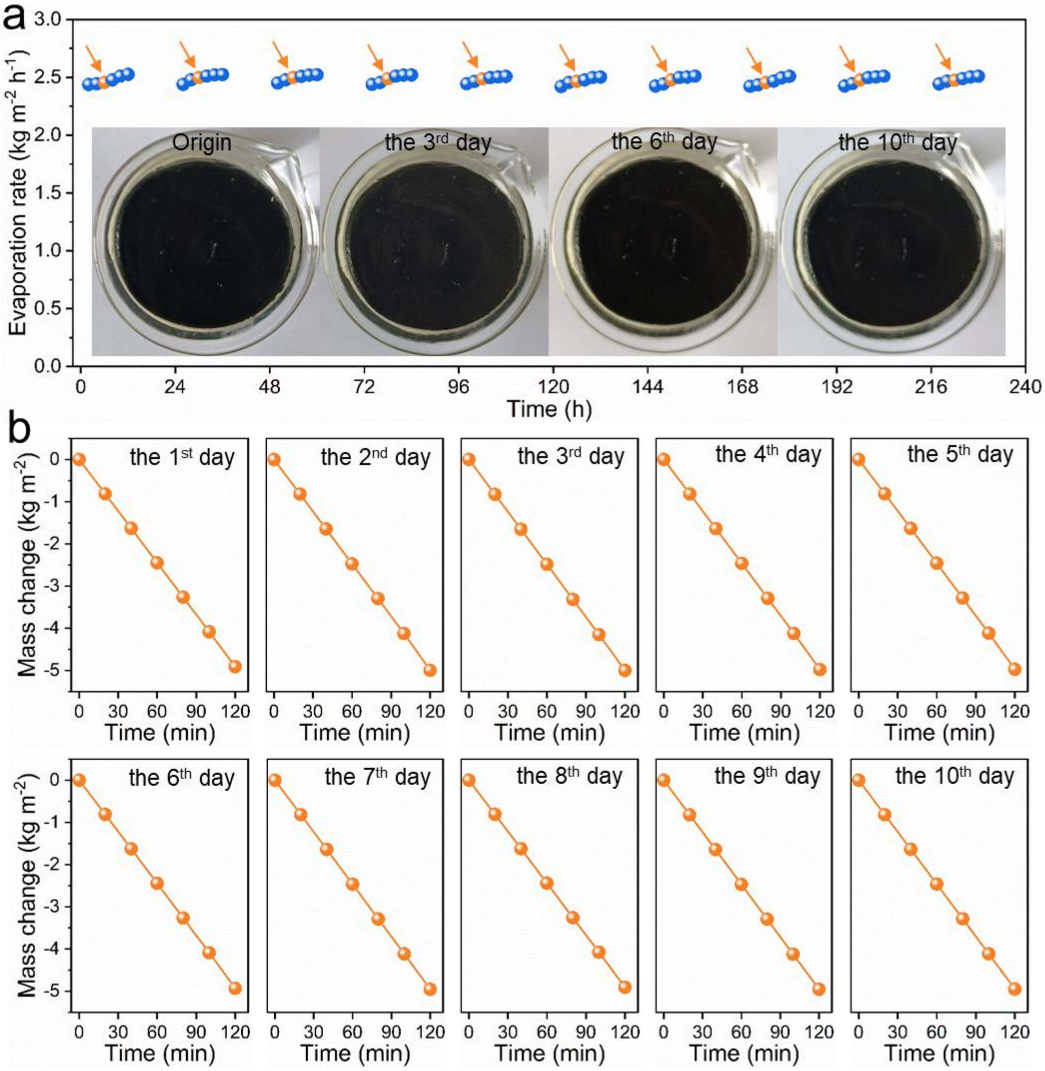

Supplementary Fig. 23. (a) Evaporation rate of seawater via CNT-hydrogel under 1 sun irradiation for 10-days, (b) Representative evaporation lines in each day. Please note: lines in (b) correspond to the orange arrows marked in (a).

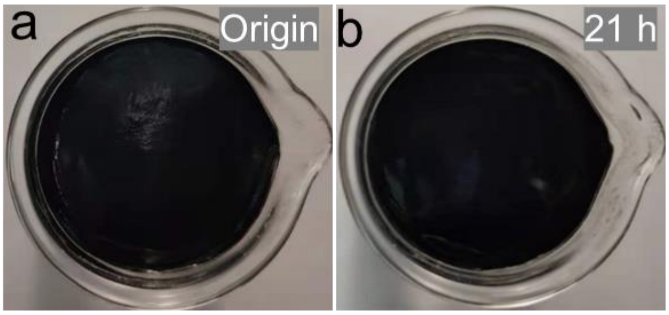

Supplementary Fig. 24. Optical photographs of the CNT-hydrogel floating on seawater (a) before and (b) after irradiation under 1 sun irradiation for 21 h.

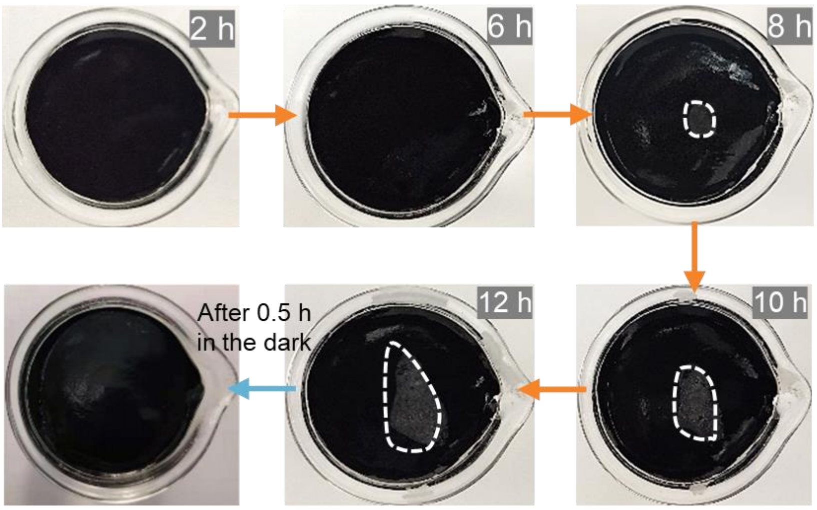

Supplementary Fig. 25. Photographs of the surface of CNT-hydrogel during the evaporation of 10wt% NaCl solution.

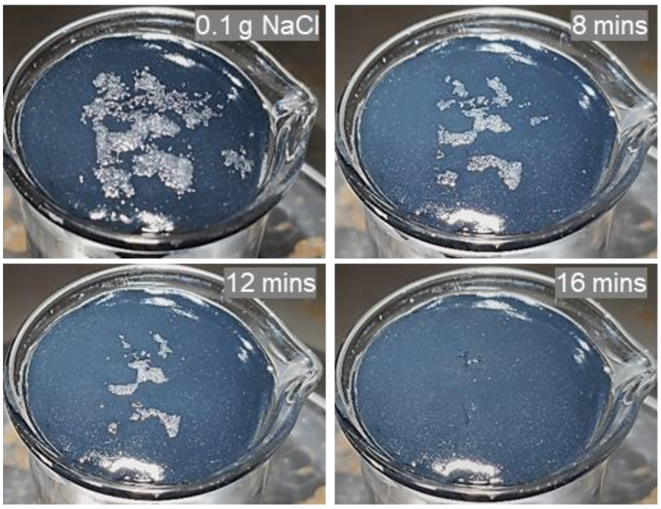

Supplementary Fig. 26. Photographs of the NaCl ablation process on the top surface of the CNT-hydrogel floating on seawater under 1 sun irradiation.

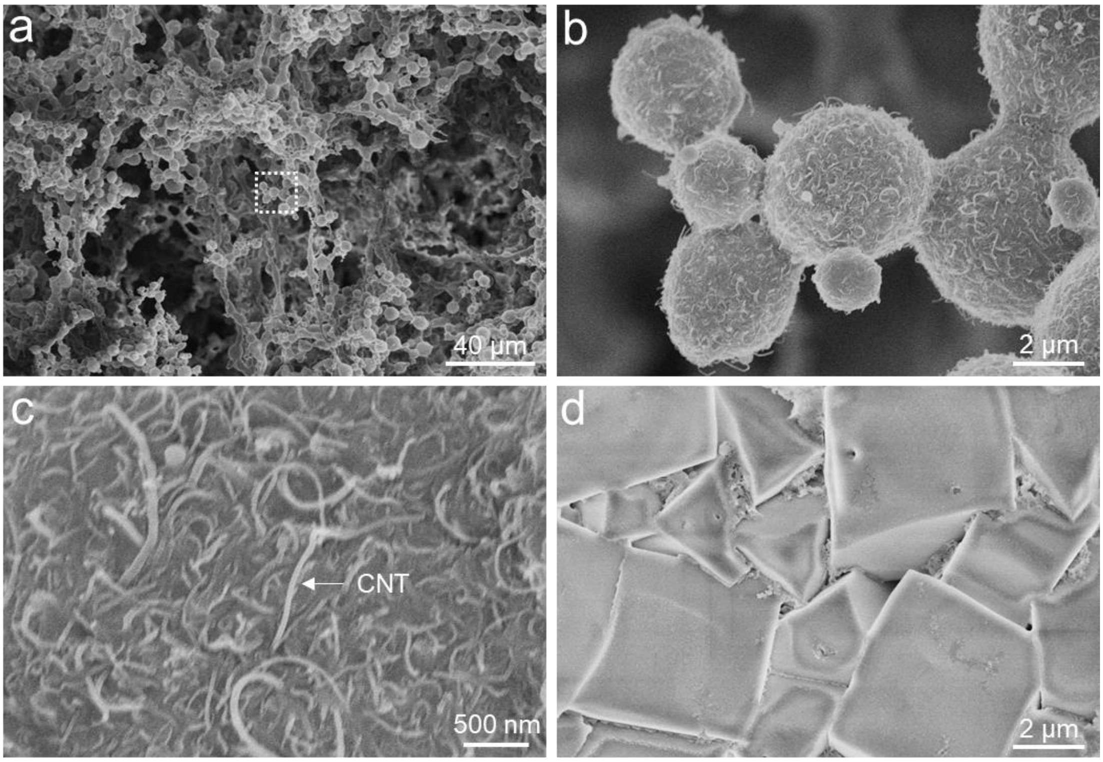

Supplementary Fig. 27. (a-c) SEM images of CNT-hydrogel after 3-days brine heat treatment (10 wt% NaCl solution, 80 ℃); (d) SEM images of salt solution dried on wafer in Figure 7(f).
