# Supplementary material for: Liquid sculpture and curing of bio-inspired polyelectrolyte aqueous two-phase systems
Source: Nat Commun. 2023 Apr 28;14:2456. doi: 10.1038/s41467-023-38236-8 (PMC10147642; doi:10.1038/s41467-023-38236-8)
Supplement: Supplementary file 3 — Description of Additional Supplementary Files [file 41467_2023_38236_MOESM3_ESM.pdf]

Description of Additional Supplementary Files

File Name: Supplementary Movie 1
Description: A video clip of PILCN1-PDDA ATPS

File Name: Supplementary Movie 2
Description: Microscopic observation of PILCN1-PDDA ATPS
